# Functional expression, purification and reconstitution of the recombinant phosphate transporter Pho89 of *Saccharomyces cerevisiae*

**DOI:** 10.1111/febs.12090

**Published:** 2013-01-28

**Authors:** Palanivelu Sengottaiyan, Lorena Ruiz-Pavón, Bengt L Persson

**Affiliations:** 1 School of Natural Sciences, Linnaeus UniversityKalmar, Sweden; 2 Laboratory of Molecular Cell Biology, Institute of Botany and MicrobiologyKU Leuven, Flanders, Belgium; 3 Department of Molecular Microbiology, Flanders Institute of Biotechnology (VIB), Leuven-Heverlee, Flanders, Belgium

**Keywords:** Pho89, phosphate transport reconstitution, *Pichia pastoris*, *Saccharomyces cerevisiae*

## Abstract

The *Saccharomyces cerevisiae* high-affinity phosphate transporter Pho89 is a member of the inorganic phosphate (Pi) transporter (PiT) family, and shares significant homology with the type III Na^+^/Pi symporters, hPit1 and hPit2. Currently, detailed biochemical and biophysical analyses of Pho89 to better understand its transport mechanisms are limited, owing to the lack of purified Pho89 in an active form. In the present study, we expressed functional Pho89 in the cell membrane of *Pichia pastoris*, solubilized it in Triton X-100 and foscholine-12, and purified it by immobilized nickel affinity chromatography combined with size exclusion chromatography. The protein eluted as an oligomer on the gel filtration column, and SDS/PAGE followed by western blotting analysis revealed that the protein appeared as bands of approximately 63, 140 and 520 kDa, corresponding to the monomeric, dimeric and oligomeric masses of the protein, respectively. Proteoliposomes containing purified and reconstituted Pho89 showed Na^+^-dependent Pi transport activity driven by an artificially imposed electrochemical Na^+^ gradient. This implies that Pho89 operates as a symporter. Moreover, its activity is sensitive to the Na^+^ ionophore monensin. To our knowledge, this study represents the first report on the functional reconstitution of a Pi-coupled PiT family member.

**Structured digital abstract:**

- Pho89 and Pho89 bind by molecular sieving (View interaction)
- Pho89 and Pho89 bind by comigration in gel electrophoresis (View interaction)
- Pho89 and Pho89 bind by molecular sieving (View interaction)
- Pho89 and Pho89 bind by comigration in gel electrophoresis (View interaction)

## Introduction

Inorganic phosphate (Pi) is an essential nutrient for almost all organisms, and is indispensable for the biosynthesis of cellular components such as nucleic acids, nucleoproteins, and phospholipids 1. In *Saccharomyces cerevisiae*, the transport of Pi across the plasma membrane can be selectively achieved by the high-affinity Pi transporters Pho84 and Pho89, which are transcriptionally regulated by the phosphate-responsive signaling pathway 1,2. Pho89 (2.A.20.2.2), an integral plasma membrane protein of 574 residues, belongs to the Pi transporter (PiT) family (Transport Classification Database number: 2.A.20), whose other members include mammalian Pit1 and Pit2 3,4, *Escherichia coli* PitA and PitB 5, *Neurospora crassa* Pho4 6, and *Arabidopsis thaliana* Pht2 7.

Pho89 functions as a high-affinity cation-dependent Pi cotransporter that plays an obligatory role in the regulation of Pi homeostasis under alkaline growth conditions 8. It shows optimal functional activity at pH 9.5, with a *K*_m_ for phosphate of 0.5 μm 9. Furthermore, the Pi transport activity of Pho89 is enhanced by the presence of Na^+^ and, to a lesser extent, Li^+^ and K^+^ 10. It has also been shown that Pho89 mediates the cotransport of one Pi with two Na^+^ per transport cycle, and that its transport activity is driven by the electrical gradient (Δ*ψ*) across the plasma membrane 11. Whole genome expression analysis has revealed that, despite its strict activation by Pi starvation, *PHO89* is also upregulated under other stress conditions, such as Mg^2+^ starvation, Ca^2+^ stress, alkaline pH, and cell wall damage 12,13.

Pho89 shows significant sequence homology with the phosphate permease Pho4 of *N. crassa* 14 and the mammalian type III Na^+^/Pi symporters, hPit1 and hPit2 15. hPit1 and hPit2 have 62% amino acid identity, and impairment of their functionality is associated with hyperphosphatemia-induced calcification of vascular tissue 16 and familial idiopathic basal ganglia calcification 17. Membrane topology prediction has revealed that Pho89 consists of 12 transmembrane domains, with both the N-termini and C-termini located at the extracellular side of the cell, and a large intracellular hydrophilic loop positioned between the seventh and eighth transmembrane domains 18.

Among the PiT family members, hPit1 and hPit2 have been well characterized at the cellular level by use of heterologous cell expression systems (fibroblast cells of murine origin and *Xenopus* *laevis* oocytes) 19,20. However, the biochemical and biophysical characterization of the PiT family members is very limited, probably because of difficulties in obtaining substantial amounts of homogeneous functional protein. Attempts to overexpress the low-affinity PiT family members PitA and PitB in an *E. coli* expression system have been unsuccessful 5. Because of its low activity and alkaline pH optimum (pH 9.5) 10, Pho89 has been difficult to characterize biochemically. In order to obtain sufficient quantities of functional protein for biochemical and biophysical analysis, we chose to use the methylotropic yeast *Pichia pastoris* for expression of Pho89.

In this study, we report the functional expression and purification of the cation-dependent PiT Pho89 with the *P. pastoris* expression system. Purified Pho89 was functionally reconstituted into proteoliposomes, and showed Na^+^ electrochemically driven Pi transport activity that could be inhibited by the Na^+^ ionophore monensin. To our knowledge, this study represents the first report on the functional reconstitution of a Pi-coupled PiT family member.

## Results and Discussion

Despite the wealth of physiological and transport studies, the biochemical and biophysical properties of PiT family members are not yet available 21–23. Investigation of these has been hampered by the inability to obtain a sufficient quantity of functional protein. An initial attempt to overexpress *Saccharomyces cerevisiae* Pho89 in the *E*. *coli*-based expression system was not successful (data not shown). Consequently, we opted to use the eukaryotic expression host *P. pastoris*, which has a number of advantages over *E*. *coli* and other eukaryotic expression hosts. *P*. *pastoris* appears to be an attractive host for recombinant protein expression, because of: (a) the ability to grow to high cell density in defined media and the ease of scaling up; (b) the presence of the very strong and tightly regulated methanol-inducible alcohol oxidase 1 promoter; and (c) the ability to perform the eukaryotic post-translational modifications and integrate the expression plasmids in its own genome in one or more specific sites by homologous recombination 24,25. Moreover, during the last few years, the *P. pastoris* expression system has been proven to be an amenable expression system for recombinant membrane protein production 26–30. Therefore, we employed this eukaryotic expression host to overproduce the full-length recombinant Pho89 fusion protein (Pho89–Myc-His_6_) in amounts suitable for biochemical and biophysical characterization of the protein.

### Optimization of Pho89 expression

**Figure 1:**
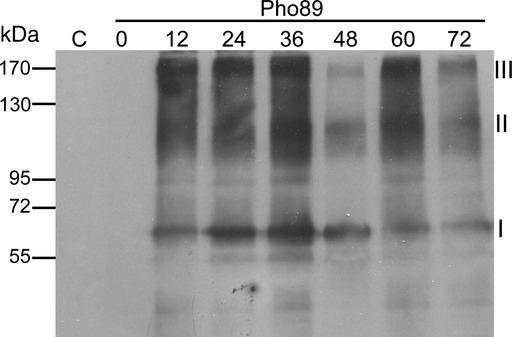
Time course for induction of *S*. *cerevisiae* Pho89 expression in *P. pastoris*. The cultures of *P. pastoris* expressing Pho89 were grown, induced, and harvested at the indicated time points (hours). Total membrane fractions were prepared as described in Experimental procedures, and 5 μg of protein was resolved by SDS/PAGE followed by western blotting with HRP-conjugated antibody against Myc. As a negative control (C), we analyzed 5 μg of the total membrane fraction from *P. pastoris* cells expressing an empty pPICZB vector. The positions of monomeric (I), dimeric (II) and oligomeric (III) species of Pho89 are indicated.

The Pho89 expression of the selected *P. pastoris* clone was monitored every 12 h for three consecutive days. After methanol induction, the total membrane fractions were prepared at the indicated time periods, and subjected to SDS/PAGE followed by western blotting with horseradish peroxidase (HRP)-conjugated antibody against Myc (Fig. 1). The signal detected at ∼ 63 kDa corresponds to the predicted molecular mass of the protein. In addition to this, high molecular mass bands could be detected at around 140 and 520 kDa, which were presumably dimeric and oligomeric forms of the protein. As a control, the total membrane fraction from *P*. *pastoris* cells transformed with an empty vector (pPICZB) was used. Time-course expression analysis revealed that the maximum expression level of Pho89 was observed after 36 h, and its expression level remained relatively constant for up to 60 h (Fig. 1). Induction for 48 h was used for subsequent functional analysis and purification of the protein.

### Functionality of Pho89 in *P*. *pastoris*

**Figure 2:**
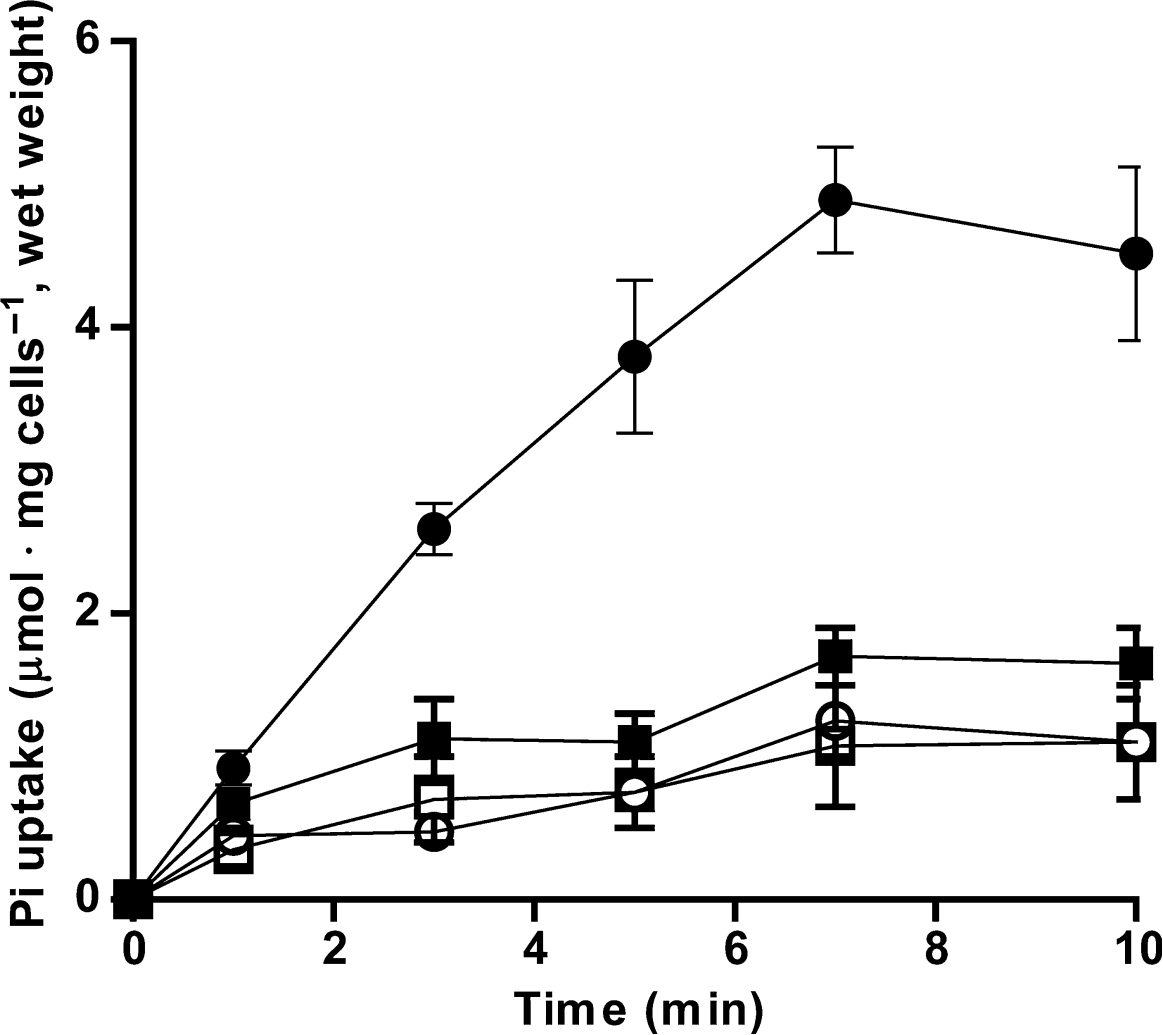
Functional expression of Pho89 in *P. pastoris*. Time-course analysis of Na^+^ dependence of Pi transport by *P. pastoris* expressing Pho89. *P*. *pastoris* cells expressing Pho89 were collected after 48 h of induction, and were prepared as described in Experimental procedures. Thirty microliters of cell suspension was incubated in uptake solution containing 25 mm Tris/succinate buffer (pH 8.0), 3% glucose and 6.25 μm [^32^P]orthophosphate in the presence (•) or absence (○) of 5 mm NaCl. Pi uptake measurements were performed at the indicated time periods. Subsequently, the reaction was stopped with ice-cold buffer, and the amount of radioactivity was measured by liquid scintillation spectrometry. Under similar conditions, Pi transport activity was measured in control cells in the presence (▪) or absence (□) of 5 mm NaCl. Data represent the mean ± SD of at least three independent experiments with four replicates.

Pho89 is an *S. cerevisiae* high-affinity Pi transporter that is upregulated under low-Pi conditions, showing a *K*_m_ for Pi of 0.5 μm 9. In these cells, half-maximal stimulation of Pi uptake was obtained in the presence of 3 mm NaCl in the assay medium, whereas close to maximal stimulation was obtained in the presence of 5 mm NaCl 9. In order to evaluate suitable conditions for assaying Pi uptake by *P. pastoris* cells harboring Pho89, a titration analysis with Pi concentrations in the range of 3.1–25 μm was carried out, and the results were compared with those obtained with cells lacking Pho89 (Fig. S1). Under conditions of 6.25 μm Pi, the Pho89-mediated uptake was four-fold to five-fold increased as compared with *P. pastoris* cells lacking Pho89. In this study, Na^+^-dependent Pi uptake measurements at pH 8.0 were performed to determine the transport activity of the expressed Pho89. As shown in Fig. 2, the Pi transport activity of the *P*. *pastoris* clone expressing Pho89 was greatly increased in the presence of NaCl (four-fold to five-fold induction), whereas no Pi uptake activity above background level could be detected in the absence of NaCl. Control samples showed only low Pi uptake activity, to a similar extent in the absence or presence of Na^+^ (Fig. 2).

These results confirmed that the *P*. *pastoris*-expressed Pho89 was functionally active, with retained electrogenic and Na^+^-dependent Pi transport activity comparable to those for Pho89 expressed in *S*. *cerevisiae* 9,10.

### Solubility screening of Pho89

**Figure 3:**
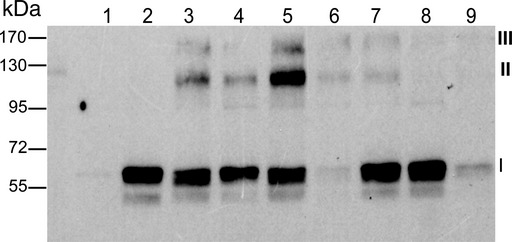
Detergent screening for solubilization of Pho89. The total membrane protein fraction was prepared as described in Experimental procedures. Fifty microliters (100 μg) of total membrane protein was diluted in 0.5 mL of resuspension buffer containing the following set of 1% detergents: lane 1, without detergent; lane 2, SDS; lane 3, C_12_E_9_; lane 4, DDM; lane 5, Triton X-100; lane 6, n-octyl-β-d-glucopyranoside; lane 7, LDAO; lane 8, foscholine-12; lane 9, Chaps. The detergent-treated membrane samples were incubated on ice for 2 h, and then centrifuged at 40 000 ***g*** for 30 min at 4 °C. Ten-microliter aliquots of the supernatants were resolved by SDS/PAGE followed by western blotting with HRP-conjugated antibody against Myc. The positions of monomeric (I), dimeric (II) and oligomeric (III) species of Pho89 are indicated.

The purification of homogeneous Pho89 from the *P*. *pastoris* cell membrane was a prerequisite for reconstitution and functional analysis. As a crucial first step, a detergent screen to extract functional Pho89 from the *P*. *pastoris* cell membrane was performed. To determine the solubilization efficiency of Pho89 from the *P*. *pastoris* cell membrane, collected membrane pellets were treated with a set of eight detergents (Fig. 3, lanes 3–9) and then immunoblotted with HRP-conjugated antibody against Myc. The efficiency of total solubilization was determined by comparing the band intensity of the Pho89 solubilized with a series of detergents (Fig. 3, lanes 3–9) with that of the denaturant SDS (Fig. 3, lane 2). Our results clearly showed that the Pho89 was maximally solubilized with Triton X-100, polyoxyethylene (8) dodecyl ether (C_12_E_9_), *N*,*N*-dimethyldodecylamine-*N*-oxide (LDAO), foscholine-12, and n-dodecyl-β-d-maltopyranoside (DDM), and minimally solubilized with n-octyl-β-d-glucopyranoside and Chaps. In addition, no Pho89 was detected in the soluble fraction in the absence of detergent (Fig. 3, lane 1). The best solubilization efficiency of Pho89 was achieved with Triton X-100 (Fig. 3, lane 5), which was therefore used for the downstream experiments. Furthermore, Triton X-100 has been successfully used for solubilization 31–33 and detergent-mediated reconstitution of several membrane proteins 33–37.

### Purification of Pho89

**Figure 4:**
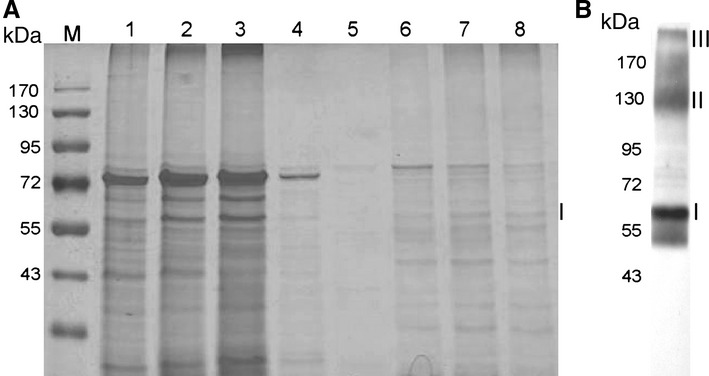
Purification of Pho89 by immobilized nickel affinity chromatography. (A) A Coomassie Brilliant Blue-stained 10% SDS/PAGE gel is shown. The lanes are as follows. M, prestained protein molecular mass marker; lane 1, aliquot of total membrane solubilized in Triton X-100; lane 2, flow-through protein from the Ni^2+^–nitrilotriacetic acid column; lane 3, column wash with column binding buffer; lane 4, column wash with binding buffer containing 50 mm imidazole; lanes 5–8, eluted fractions of Pho89. (B) Aliquots from pooled eluted fractions were examined by western blotting with HRP-conjugated antibody against Myc. The positions of monomeric (I), dimeric (II) and oligomeric (III) species of Pho89 are indicated. All samples were treated with 2× urea loading sample loading buffer at 37 °C for 30 min before analysis.

To purify Pho89 from *P*. *pastoris*, the protein was solubilized from the cell membrane with 1% Triton X-100 or 1% foscholine-12, and subjected to purification by metal affinity chromatography. Pho89 bound to the column resin was eluted with 250–300 mm imidazole. The eluted Pho89 fractions were monitored by SDS/PAGE with Coomassie Brilliant Blue staining (Fig. 4A) and by western blotting with an HRP-conjugated antibody against Myc, which revealed a prominent band with an apparent molecular mass of 63 kDa (Figs 4B and S2A). Furthermore, we also detected high molecular mass bands at approximately 140 and 520 kDa that probably correspond to dimeric and oligomeric forms of the protein (Figs 4B and S2A).

**Figure 5:**
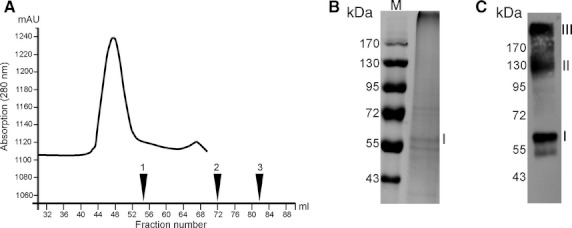
Gel filtration purification of Pho89. (A) Pho89 solubilized in Triton X-100 and purified by Ni^2+^ affinity chromatography was further purified on a HiLoad 16/60 Superdex 200 prep grade column, and fractions were collected as described in Experimental procedures. Arrows indicate the positions of the eluted standard proteins ferritin (1), catalase (2), and aldolase (3), with molecular masses of 440, 232 and 158 kDa, respectively. Elution of all proteins was followed by absorbance at 280 nm. An aliquot of purified Pho89 was treated with 2× urea loading sample buffer at 37 °C for 30 min, and separated by 10% SDS/PAGE followed by Coomasie Brilliant Blue staining (B) and western blotting analysis with HRP-conjugated mouse antibody against Myc tag (C). The positions of monomeric (I), dimeric (II) and oligomeric (III) species of Pho89 are indicated.

Size exclusion chromatography was used to assess the oligomeric state of the detergent-solubilized and purified Pho89. The size exclusion profile showed that Pho89 was eluted as a single peak from the gel filtration column, as shown in Fig. 5A. Relative to protein standards, our results clearly indicate that purified Pho89 was in a 520-kDa oligomeric form. Analysis of the oligomer subjected to SDS/PAGE (Figs 5B and F S2B), followed by western blotting analysis, revealed that the signal corresponded to the size of the 63-kDa monomer, the 140-kDa dimer and the 520-kDa oligomeric species of Pho89 (Figs 5C and S2C). These results indicate that the oligomeric form of Pho89 was partially resistant as well as being dissociated into dimeric and monomeric forms upon treatment with SDS. Furthermore, our results imply that purified Pho89 remained in the oligomeric state when the protein was treated with other detergents (e.g. foscholine-12), indicating that the protein was mainly present as the oligomeric form in the detergent solution. Previous studies have shown that the homologous protein, human Pit2, also forms homo-oligomers in the cell membrane 38 that are partly resistant to SDS 31,39. There has been increasing evidence that many transporters function as oligomers 40–43. Indeed, Salaün *et al*. 31 showed that human Pit2 functions as a homodimer. Moreover, treatment of purified Pho89 with dithiothreitol (100 mm) does not affect dimer/oligomer formation (Fig. S3A,B). These results support the notion that disulfide bonds are not involved in dimer/oligomer formation 31. The reason for the improved detection of the protein bands seen at 130 and 170 kDa, possibly representing the dimeric and trimeric forms of the protein, with the antibody against Myc but not with the antibody against His is, at present, not clear. The western blotting analysis also indicated that the band of lower molecular mass than Pho89 was a degradation product of Pho89 (Fig. S3A,B).

### Functional reconstitution of Pho89 – assessment of Pi transport

After the two-step purification, we obtained ∼ 75 μg·mL^−1^ purified recombinant Pho89 per liter of culture. We investigated the functionality of purified Pho89 by reconstitution into liposomes with a detergent-mediated reconstitution procedure, as previously established for Pho84 33. The incorporation of purified recombinant Pho89 into liposomes was verified by western blotting analysis (Fig. S4, lanes 3 and 4). This result suggests that purified recombinant full-length Pho89 exists in oligomeric form both in detergent solution and, probably, also in the lipid bilayer.

**Figure 6:**
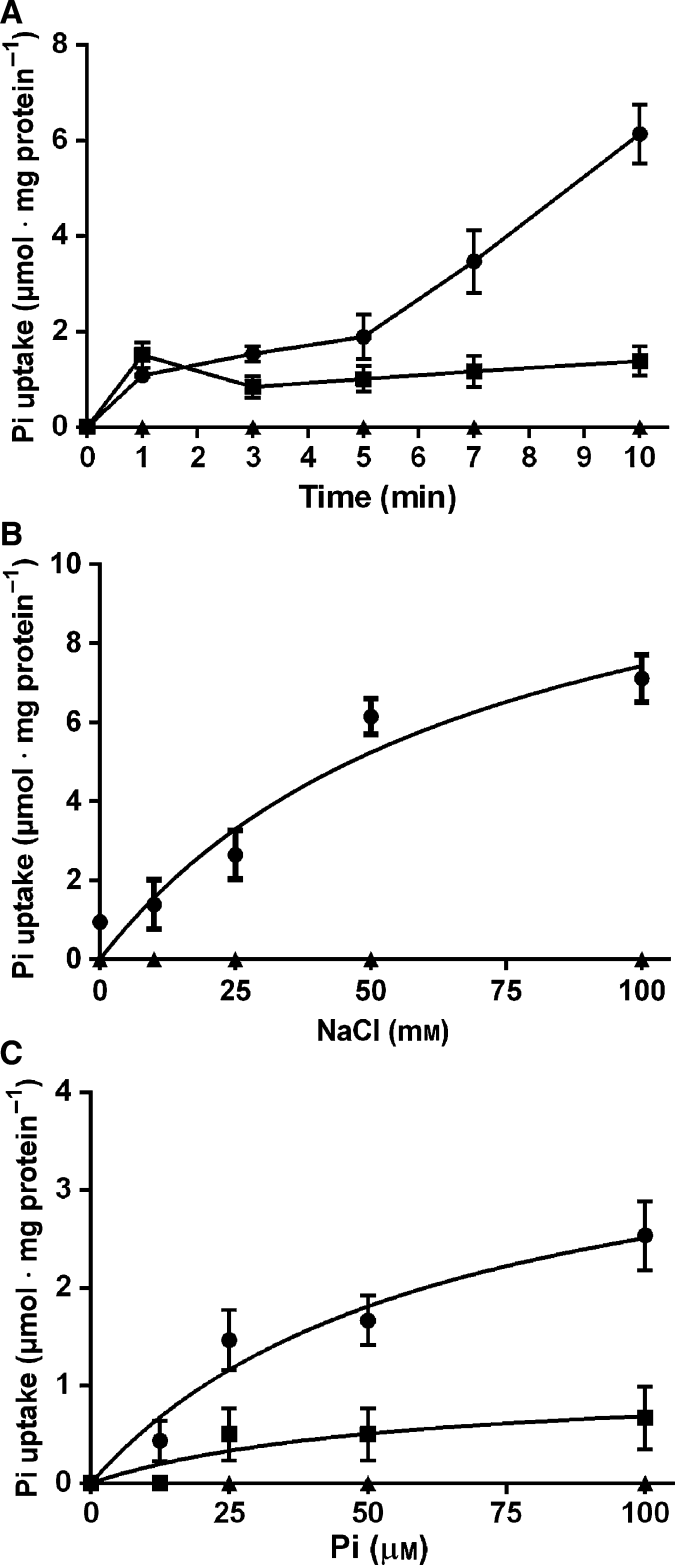
Biochemical characterization of Pho89 in reconstituted proteoliposomes. (A) Na^+^ dependence of Pi transport. The reconstituted proteoliposomes were preloaded with 25 mm Hepes-K^+^ buffer at pH 7.0, and diluted 25-fold in assay buffer (25 mm Tris/succinate, pH 10) in the presence (•) or absence (▪) of 50 mm NaCl. Transport was initiated by addition of 100 μm ^32^P. At the times indicated, transport was stopped, and the samples were assayed by rapid filtration and liquid scintillation spectroscopy. (B) Na^+^ concentration dependence of Pi transport. The reconstituted proteoliposomes were preloaded under the same conditions as in (A), in the presence of the indicated concentration of Na^+^. (C) Dose dependence curve of Pi transport. The reconstituted proteoliposomes were preloaded under the same conditions as in (A) and (B), in the absence (▪) or presence (•) of 50 mm NaCl, plus ^32^P at the concentrations indicated (0–100 μm). All of the experiments where perfomed under similar conditions in control liposomes without Pho89 (▲). Data represent the mean ± SD of at least three independent experiments with four replicates.

Pho89 in *S. cerevisiae* cells has previously been shown to be active at alkaline pH, and is highly specific for Na^+^ at concentrations from 5 to 25 mm 9,10. On the basis of our current analysis of Pi uptake by Pho89 in proteoliposomes, 50 mm NaCl was used for the ΔpNa^+^-driven uptake studies described here. As shown in Fig. 6A, the proteoliposomes were able to catalyze accumulation of ^32^P upon energization. The level of ^32^P accumulation was dependent on the Δp generated in the presence of Na^+^, as the rate of Pi uptake was increased approximately four-fold to five-fold after 10 min of incubation. No significant uptake could be observed in the absence of Na^+^. In contrast to the Pho89-mediated Pi uptake in intact yeast cells, where maximal three-fold activation was seen in the presence of 25 mm NaCl 9, maximal activation of Pi uptake into the well-sealed proteoliposomes appears to require a higher Na^+^ gradient, with up to 100 mm in the assay medium (Fig. 6B).

**Figure 7:**
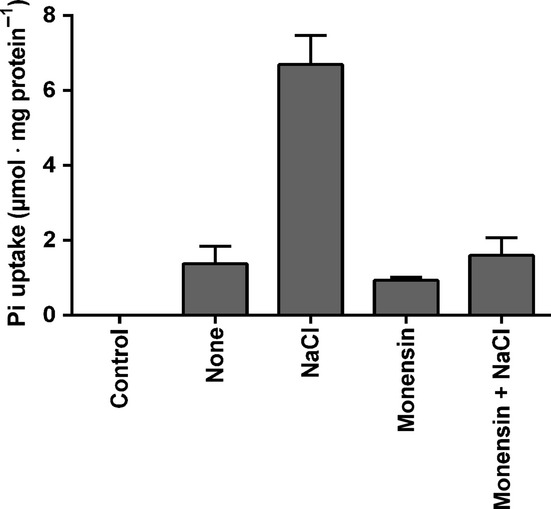
Inhibition of uptake of Pi into proteoliposomes containing purified Pho89. The reconstituted proteoliposomes were preloaded with 25 mm Hepes-K^+^ buffer at pH 7.0, and diluted 25-fold into 25 mm Tris/succinate (pH 10) in the absence or presence of 50 mm NaCl. The above proteoliposome mix was preincubated for 2 min in the absence or the presence of 100 μm monensin. Transport was initiated by addition of 100 μm ^32^P. After 10 min, the transport was stopped as described in Experimental procedures. Pi transport activity was measured in control liposomes containing 50 mm NaCl and 100 μm monensin. Data represent the mean ± SD of at least three independent experiments with four replicates.

Next, we investigated the effect of Pi concentration on the ^32^P uptake mediated by proteoliposomes containing Pho89. In the presence of ΔpNa^+^, the Pi concentration dependence showed hyperbolic behavior (Fig. 6C), with an apparent *K*_m_ of 64.1 ± 23.3 μm and a *V*_max_ of 4.10 ± 0.77 μmol·min^−1^·(mg protein)^−1^. These values are in agreement with the recognition of Pho89 as a high-affinity transporter. To determine whether Pi uptake by the reconstituted proteoliposomes directly depends on ΔpNa^+^, the proteoliposomes were preincubated with 100 μm of the Na^+^ ionophore monensin for 2 min prior to measurement of the Pi transport activity. As shown in Fig. 7, in the presence of monensin, Pi transport was reduced six-fold to seven fold as compared with Pi transport in the presence of Na^+^. Altogether, these results reinforce the identity of Pho89 as an Na^+^-dependent Pi symporter.

## Conclusion

In summary, we have succeeded in expressing and purifying the full-length *S*. *cerevisiae* high-affinity PiT Pho89 by using a *P. pastoris*-based expression system. The recombinant Pho89 retained its functionality when expressed in the intact cell system and when the purified protein was reconstituted into proteoliposomes. In conclusion, functionally active Pho89 utilizes ΔpNa^+^ as a driving force for Pi transport, and operates with Na^+^ via a symport mechanism. This study represents the first characterization of a Pi-coupled PiT family member in a reconstituted system, and provides a basis on which to perform detailed biochemical, biophysical and structural analyses of this family of transporters.

## Experimental procedures

### Materials and strains

The *P*. *pastoris* wild-type strain X-33, pPICZB vector, Zeocin antibiotic and Yeast Nitrogen Base medium (without amino acids and with ammonium sulfate) were purchased from Invitrogen (Carlsbad, CA, USA). The HRP-conjugated mAb against Myc and the HRP-conjugated mAb against His C-terminus were from Invitrogen (Carlsbad, CA, USA). Phenylmethanesulfonyl fluoride and monensin were obtained from Sigma (St. Louis, MO, USA). [^32^P]Orthophosphate (carrier-free) was from Perkin Elmer (Waltham, MA, USA). Complete EDTA-free protease inhibitor cocktail tablets were from Roche Diagnostics (Basel, Switzerland). The HisTrap chelating column was from GE Healthcare (Pittsburgh, PA, USA). The detergents C_12_E_9_, LDAO and foscholine-12 were from Anatrace (Maumee, OH, USA). The lipids and Bio-Beads (SM-2) were from Lipid Products (Redhill, Surrey, UK) and Bio-Rad (Hercules, CA, USA), respectively.

### Cloning and heterologous expression of Pho89 in *P. pastoris*

The *S. cerevisiae PHO89* gene was PCR-amplified from the pU6H2MYC vector 44 with the forward primer 5′-TTAAGCAAAGAATTCATGGCTTTACATCAATTTGAC-3′ and the reverse primer 5′-GAGAGAGCGGCCGCTGTCATTTGGTATTCCACACC-3′ containing *Eco*RI and *Not*I restriction sites (underlined), respectively. The PCR product and pPICZB vector were digested with *Eco*RI and *Not*I, and ligated together to obtain *PHO89* fused with the Myc epitope followed by a His_6_ tag at its C-terminus. The resulting pPICZB–*PHO89*–c-Myc-His_6_ construct was verified by DNA sequencing. Approximately 10 μg of linearized plasmid DNA of pPICZB–*PHO89*–c-Myc-His_6_ and pPICZB vector (control) constructs were obtained by digestion with *Sac*I and were transformed into *P*. *pastoris* electrocompetent cells (wild-type strain X-33) with the electroporation method described in the Invitrogen *P. pastoris* expression kit manual. The transformants were initially selected on yeast extract peptone dextrose sorbitol agar plates supplemented with 100 μg·mL^−1^ Zeocin. Then, multiple-copy transformants were further screened by the use of yeast extract peptone dextrose sorbitol agar resistance plates containing Zeocin at final concentrations of 0.5, 1 and 2 mg·mL^−1^. The clones resistant to growth at higher Zeocin concentration (2 mg·mL^−1^) were selected for screening of Pho89 expression.

### Screening of *P. pastoris* clones for Pho89 expression

Clones capable of growing in 2 mg·mL^−1^ Zeocin were chosen for small-scale (50 mL) expression screening of Pho89. For the initial protein expression screening analysis, each of the *P*. *pastoris* clones carrying the pPICZB–*PHO89*–c-Myc-His_6_ construct, and the clone carrying an empty pPICZB plasmid (control), were grown separately in 10 mL of buffered glycerol-complex medium (BMGY) (1% yeast extract, 2% peptone, 100 mm potassium phosphate, pH 6, 1.34% Yeast Nitrogen Base, 4 × 10^−5^% biotin, 1% glycerol) agitated at 200 r.p.m. for 18 h at 30 °C, and subsequently pelleted by centrifugation at 2000 ***g*** for 5 min at room temperature. The initiation of protein expression was achieved by resuspending the pelleted cells in 50 mL of buffered methanol-complex medium (BMMY) (same as BMGY, except that it contained 1% methanol in place of glycerol) to obtain an *A*_600 nm_ of 1.0, followed by growth at 200 r.p.m. at 30 °C for 48 h. The cells had already been induced, so the addition of methanol (1% v/v) served to maintain the induction, as described later. Cells were harvested by centrifugation for 10 min at 1500 ***g*** at 4 °C. Typically, an average of 17–18 g of cells (wet pellet) was harvested per liter of culture. The cell pellets were frozen, and stored at −80 °C prior to western blotting analysis.

### Preparation of cell membrane from *P. pastoris* clones and immunoblotting

For protein expression analysis, the frozen cell pellets were thawed on ice and washed once with ice-cold wash buffer (50 mm sodium phosphate buffer, pH 7.5, 500 mm NaCl, 10% glycerol, 1 mm phenylmethanesulfonyl fluoride). Each cell pellet was resuspended in an equal volume of ice-cold lysis buffer (50 mm sodium phosphate buffer, pH 7.5, 500 mm NaCl, 10% glycerol, 1 mm phenylmethanesulfonyl fluoride) supplemented with protease inhibitor cocktail (complete EDTA-free) and glass beads (cell pellet/ice-cold lysis buffer/glass beads at a ratio of 1 : 1 : 1). The cells were lysed by vortexing for 8 × 1-min cycles, with 1 min incubation on ice between the cycles. The cell lysate was centrifuged at 500 ***g*** for 10 min at 4 °C, and the resulting supernatant was further centrifuged at 40 000 ***g*** for 90 min at 4 °C. The membrane pellet (total membrane fraction) was resuspended in ice-cold resuspension buffer (50 mm Tris/HCl, pH 8.0, 500 mm NaCl, 10% glycerol, 1 mm phenylmethanesulfonyl fluoride) supplemented with protease inhibitor cocktail (complete EDTA-free). The protein content of the total membrane fraction was measured with the Bradford assay method, with BSA as standard. Five micrograms of total membrane fractions from each clone was mixed with 2× urea sample loading buffer (120 mm Tris/HCl, pH 6.8, 4% SDS, 20% glycerol, 4 m urea, and 4 μg of bromophenol blue), and incubated at 37 °C for 30 min. The samples were resolved by 10% SDS/PAGE, and blotted onto a poly(vinylidene difluoride) membrane (Immobilon-P; Millipore, Billerica, MA, USA) according to the western blotting protocol (Amersham Bioscience, Piscataway, NJ, USA). The blot was blocked in 25 mL of blocking buffer (20 mm Tris/HCl, pH 7.4, 150 mm NaCl, 0.1% Tween-20) with 5% skimmed milk powder overnight at 4 °C, and this was followed by three washes with the blocking buffer. After incubation of the blot with blocking buffer, the HRP-conjugated antibody against Myc was used at a dilution of 1 : 5000 in blocking buffer containing 5% skimmed milk powder. The blot was visualized with an enhanced chemiluminescence kit, according to the manufacturer's instructions (Amersham Biosciences).

### Time-course expression analysis of recombinant Pho89

The clone showing the highest Pho89 expression was selected for further time-course expression analysis. For the time-course expression analysis, the selected clone expressing Pho89 was grown on BMGY for 18 h, and pelleted by centrifugation at 2000 ***g*** for 5 min. To induce protein expression, the pelleted cells resuspended in 50 mL of BMMY to an *A*_600 nm_ of 1.0 were grown at 30 °C for up to 72 h. In order to maintain the inducing environment, methanol was added every 24 h to a final concentration of 1% (v/v). Cells were harvested after the indicated time periods, and the total membrane fractions were prepared according to the procedure described above. The total membrane fractions (5 μg) were subjected to 10% SDS/PAGE followed by western blotting analysis with HRP-conjugated antibody against Myc, as previously described.

### Assay for Na^+^ dependence of Pi uptake in *P*. *pastoris* expressing Pho89

After induction under optimized expression conditions, the *P. pastoris* clone expressing Pho89 and the control (clone carrying an empty pPICZB plasmid) cells were grown in BMMY for 48 h, as described above. The pelleted cells were washed twice with 25 mm Tris/succinate buffer (pH 8.0), collected at 1500 ***g*** for 10 min, and subsequently resuspended in uptake buffer consisting of 25 mm Tris/succinate (pH 8.0) and 3% glucose, in the presence or absence of 5 mm NaCl. The uptake was initiated by mixing 30 μL of cell suspension with 1 μL of uptake solution containing [^32^P]orthophosphate (carrier-free, 0.18 Ci·μmol^−1^; 1 mCi = 37 MBq) to a final concentration of 6.5 μm. The cell suspension was incubated at room temperature for the indicated time periods. Pi transport was terminated by the addition of 1 mL of ice-cold 25 mm Tris/succinate buffer (pH 8.0) followed by filtration of the cell suspension as described previously 10. After three additional washes with the same ice-cold buffer, the radioactivity retained on the filter was determined by liquid scintillation spectrometry. The clone carrying an empty pPICZB plasmid (control) was used to measure endogenous transport activity.

### Solubilization trials

The cells were harvested after 48 h of induction, and the total membrane fractions were prepared as described above. The prepared total membrane was resuspended in ice-cold resuspension buffer (supplemented with protease inhibitor) at a protein concentration of ∼ 2 mg·mL^−1^. For small-scale solubilization trials, aliquots of resuspended membrane were diluted five-fold with resuspension buffer containing the following set of detergents at 1% on the membrane suspension. The nonionic detergents were C_12_E_9_, DDM, Triton X-100, and n-octyl-β-d-glucopyranoside, and the zwitterionic detergents were LDAO, foscholine-12, and Chaps. The protein detergent mixtures were incubated on ice for 2 h with mild agitation, after which insoluble material was removed by centrifugation at 40 000 ***g*** for 30 min at 4 °C. The solubilized material (supernatant) was resolved by 10% SDS/PAGE, and analyzed by immunoblotting with HRP-conjugated antibody against Myc. A sample solubilized with buffer containing 1% (w/v) SDS was used as a positive control.

### Recombinant Pho89 purification

For purification, Pho89 was solubilized and maintained in solution with Triton X-100 detergent. After 48 h of induction, the total cell membrane was prepared and solubilized with the resuspension buffer containing 1% Triton X-100 or foscholine-12. The mixture was incubated on ice for 2 h with mild agitation. After centrifugation (40 000 ***g*** for 30 min at 4 °C), the supernatant was filtered (0.45-μm filter) and applied onto a His-Trap Ni^2+^ affinity column (GE Healthcare, USA). The column had previously been equilibrated with the column equilibration buffer (50 mm Tris/HCl, pH 8.0, 500 mm NaCl, 10% glycerol, 30 mm imidazole, and 0.2% Triton X-100 or 0.1% foscholine-12). The unbound protein was washed out with the column equilibration buffer containing 50 mm imidazole, after which bound His_6_-tagged Pho89 was eluted with a linear gradient of imidazole (final concentration of 1000 mm). The major proportion of the bound Pho89 was eluted in the presence of 250–300 mm imidazole.

The eluted fractions (0.7-mL fractions) containing Pho89 (as determined by SDS/PAGE followed by western blotting analysis) were pooled, and further purified by gel filtration chromatography on a HiLoad 16/60 Superdex 200 prep grade column (GE Healthcare, Pittsburgh, PA, USA). The column was pre-equilibrated with gel filtration buffer (Tris/HCl, pH 8.0, 200 mm NaCl, 10% glycerol, and 0.2% Triton X-100 or 0.1% foscholine-12), and the gel filtration was performed at a flow rate of 1 mL·min^−1^. Size calibration was carried out with standard proteins: ferritin, 440 kDa; catalase, 232 kDa; and aldolase, 158 kDa (the corresponding elution volumes are marked by arrows in Fig. 5). Elution was followed by monitoring the absorbance at 280 nm. The purity of the recombinant Pho89 was estimated by analysis of aliquots on 10% SDS polyacrylamide gels stained with Coomassie Brilliant Blue R-250. The fractions corresponding to Pho89 were identified by western blotting with HRP-conjugated antibody against Myc.

The Pho89-enriched fractions were pooled together for reconstitution into lipid vesicles. To further assess whether disulfide bonds were involved in oligomer formation, purified Pho89 (12 μL) was incubated with 1× SDS sample loading buffer (50 mm Tris/HCl, pH 6.8, 2% SDS, 10% glycerol, 0.04% bromophenol blue) in the absence or presence of 100 mm dithiothreitol. The samples were incubated at 65 °C for 10 min prior to loading, and subjected to SDS/PAGE separation followed by western blotting analysis with HRP-conjugated antibodies against Myc and His (C-terminus).

### Reconstitution of purified Pho89

Purified Pho89 was incorporated into liposomes via a detergent-mediated reconstitution method, as described previously 33. Briefly, the liposomes were made of a mixture of phosphatidylcholine (42.5% w/v), phosphatidylethanolamine (42.5% w/v), lysophosphatidylcholine (10% w/v), and phosphatidylserine (5% w/v). The lipids were transferred into a glass screw-cap tube, and dried with nitrogen gas to obtain a thin layer of dry lipids. Subsequently, the dried lipid mixture was redissolved in diethyl ether, washed with ethanol, and dried again. The above mixture was suspended to a final concentration of 20 mg·mL^−1^ in buffer containing 25 mm Hepes-K^+^ (pH 7.0) and 0.1% Triton X-100, and sonicated in a bath-type sonicator until clear. Detergent-destabilized liposomes (1 mL) were mixed with purified Pho89 (0.05 mg·mL^−1^), and incubated for 30 min at 4 °C with gentle shaking. Proteoliposomes were formed upon removal of the Triton X-100 by incubating the above lipid/protein suspension with wet SM-2 Bio-beads (100 mg·mL^−1^) that had been prepared as previously described 45. After 2 h of mixing at 4 °C, Bio-beads were removed by centrifugation (12 000 ***g*** for 1 min), and the sample was supplemented with additional Bio-beads (150 mg·mL^−1^) and incubated for 2 h; the procedure was repeated, and this was followed by overnight incubation (12–14 h) at 4 °C. The turbid proteoliposome suspension was diluted four-fold with ice-cold 25 mm Hepes-K^+^ (pH 7.0) and pelleted by centrifugation at 247 000 ***g*** for 40 min at 4 °C. The pellet was resuspended in 0.2 mL of 25 mm Hepes-K^+^ (pH 7.0), and used for the transport assay measurements. The control liposomes were prepared similarly, except that Pho89 was omitted.

### Proteoliposomal Pi transport assay

Pi uptake was assayed in reconstituted proteoliposomes containing Pho89 and in control liposomes (without protein), and was energized by an artificial sodium gradient (ΔpNa^+^). To create an artificial ΔpNa^+^, 2 μL of the proteoliposomes was diluted 25-fold into assay buffer (25 mm Tris/succinate, pH 10), in the absence and presence of 50 mm NaCl. Pi uptake was assayed by the addition of 2 μL of [^32^P]orthophosphate (carrier-free, 50 mCi·mmol^−1^; 1 mCi = 37 MBq; Perkin Elmer) to a final concentration of 100 μm. The suspension was incubated at 25 °C for the indicated time periods. Pi transport was terminated by the addition of 1 mL of ice-cold assay buffer, and rapid filtration (using the filter type Supor-200, 0.2 μm; Gelman Sciences, Ann Arbor, MI, USA) 46 under vacuum, and this was followed by three washes with the same ice-cold buffer. The radioactivity retained on the filters was determined by liquid scintillation spectrometry.

When the effect of Na^+^ concentration on Pi uptake was measured, the amount of Pi was kept constant (100 μm), and the amount of NaCl added to the reaction mixture was varied (0–100 mm). For uptake inhibition experiments, proteoliposomes were preincubated for 2 min with the Na^+^ ionophore monensin (at a concentration of 100 μm) before the addition of 100 μm ^32^P. The uptake data are presented as the mean ± standard deviation (SD) of at least three independent experiments with four replicates, and the datasets were processed with graphpad prism.

## Abbreviations

BMGY: buffered glycerol-complex medium
BMMY: buffered methanol-complex medium
C_12_E_9_: polyoxyethylene (8) dodecyl ether
DDM: n-dodecyl-β-d-maltopyranoside
HRP: horseradish peroxidase
LDAO: *N*,*N*-dimethyldodecylamine-*N*-oxide
Pi: inorganic phosphate
PiT: inorganic phosphate transporter
SD: standard deviation
ΔpNa^+^: electrochemical Na^+^ concentration gradient

## Supporting Information

**Fig. S1.** Kinetics of phosphate uptake by *P. pastoris* cells expressing Pho89 from *S. cerevisiae*.

**Fig. S2.** Purification of Pho89, with foscholine-12 used for solubilization and purification.

**Fig. S3.** Immunoblot analysis of purified Pho89 under nonreducing (without dithiothreitol) and reducing (with dithiothreitol) conditions.

**Fig. S4.** Western blot analysis of reconstituted Pho89 in proteoliposomes.
