# Supplementary material for: Functional expression, purification and reconstitution of the recombinant phosphate transporter Pho89 of Saccharomyces cerevisiae
Source: FEBS J. 2013 Jan 28;280(3):965–75. doi: 10.1111/febs.12090 (PMC3633241; doi:10.1111/febs.12090)
Supplement: Supplementary file 1 [file febs0280-0965-SD1.zip › febs12090-sup-0001-FigsS1-S4.pdf]

the FEBS Journal
www.febsjournal.org

Functional expression, purification and reconstitution of the recombinant phosphate transporter Pho89 of *Saccharomyces cerevisiae*

Palanivelu Sengottaiyan, Lorena Ruiz-Pavón and Bengt L. Persson

DOI: 10.1111/febs.12090

Supplementary material

Fig. S1

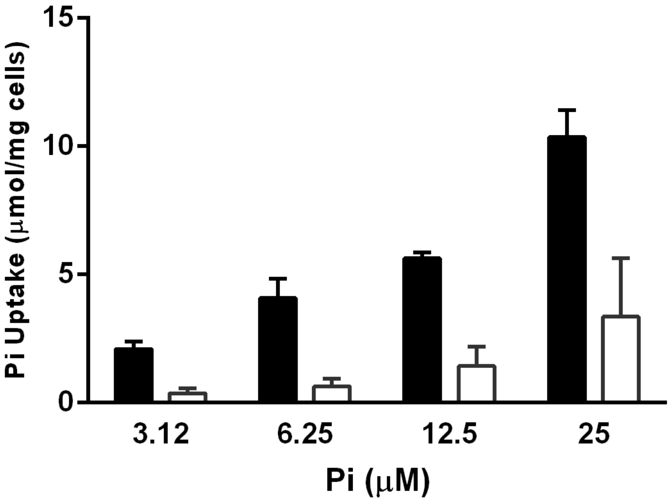

Fig. S1. Kinetics of phosphate uptake by *P. pastoris* cells expressing Pho89 from *S. cerevisiae* (■). The cells were collected after 48 h post induction and were prepared as described in Material and methods. 30 μl of cell suspension was incubated in uptake solution containing 25 mM Tris-succinate (pH 8.0), 3% glucose, 5 mM NaCl and varied concentration of [^{32}P] orthophosphate. After 10 min, the reaction was stopped using ice-cold buffer and the amount of radio activity was measured by liquid scintillation spectrometry. Under the similar conditions, control cell samples (without Pho89 protein) was measured for phosphate transport activity (❑).

Fig. S2

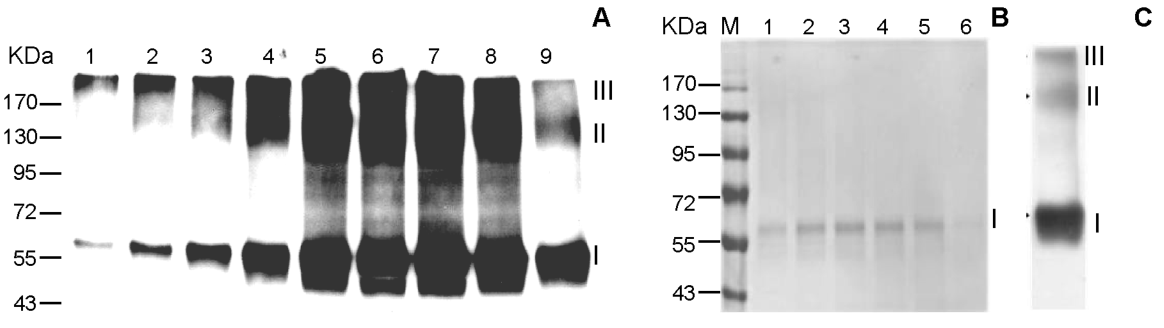

Fig. S2. Purification of Pho89, with foscholine-12 for solubilization and purification. A. The cell membrane containing Pho89 protein was solubilized in Foscholine-12 and purified by Ni^{2+} affinity chromatography. An aliquot of Ni^{2+} affinity purified fractions (lanes 1-9) were resolved on SDS-PAGE followed by Western blot analysis.

B. Purification of Pho89 from pooled Ni^{2+} affinity fractions by gel-filtration chromatography. Coomassie brilliant blue stained SDS-polyacrylamide gel of the collected fractions (lanes 1-6) followed by Western-blot analysis (C). All the samples were treated with the 2x urea loading sample buffer at 37 ºC for 30 min before analysis. The Western blot analysis was carried out with anti-myc HRP linked antibody. The positions of monomeric (I), dimeric (II) and oligomeric (III) species of the Pho89 protein are indicated.

Fig. S3

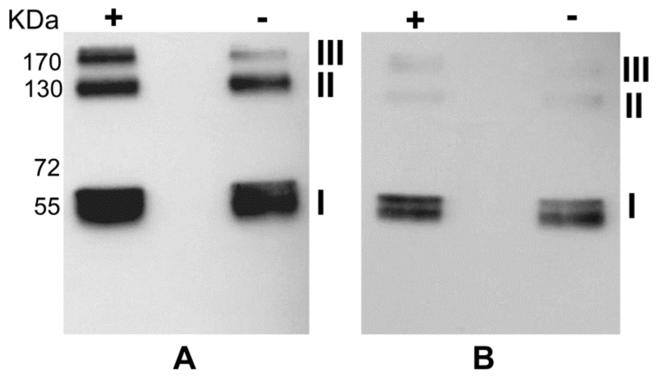

Fig. S3. Immunoblot analysis of purified Pho89 under nonreducing (without dithiothreitol) and reducing (with dithiothreitol) conditions. The purified Pho89 protein was treated with sample loading buffer in the absence (**—**) or presence (+) of 100 mM DTT at 65 ºC for 10 min and subjected to SDS-PAGE, followed by Western blot analysis with anti-myc HRP linked antibody (A) and anti-His (C-term) HRP antibody (B). The positions of the Pho89 monomer (I), dimer (II) and oligomer (III) bands are indicated.

Fig. S4

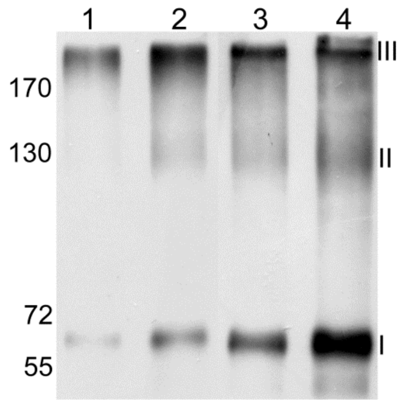

Fig. S4. Western blot analysis of reconstituted Pho89 in proteoliposomes. Pooled Pho89 containing gel filtration fractions (lane 1, 0.4 µg protein; lane 2, 0.8 µg protein) were reconstituted into liposomes as described in *Materials and Methods*. The presence of purified Pho89 in proteoliposomes (lane 3, 0.8 µg protein; lane 4, 1.6 µg protein) was detected by Western blot analysis using anti-myc HRP linked antibody. All samples were treated with 2X urea sample loading buffer at 37 ºC for 30 min before analysis. The positions of the Pho89 monomer (I), dimer (II) and oligomer (III) bands are indicated.
